# Comparison of clinical and radiological outcomes between opening-wedge and closing-wedge high tibial osteotomy: A comprehensive meta-analysis

**DOI:** 10.1371/journal.pone.0171700

**Published:** 2017-02-09

**Authors:** Lingfeng Wu, Jun Lin, Zhicheng Jin, Xiaobin Cai, Weiyang Gao

**Affiliations:** 1 Department of Orthopedics, the Fifth Affiliated Hospital & Central Hospital of Lishui City of Wenzhou Medical University, Lishui, China; 2 Department of Orthopedics, Qingyuan Country People’s Hospital, Lishui, China; 3 Department of Surgery, Second Clinical Medical College, Wenzhou Medical University, Wenzhou, China; 4 Department of Orthopedics, the Second Affiliated Hospital & Yuying Children’s Hospital of Wenzhou Medical University, Wenzhou, China; Harvard Medical School/BIDMC, UNITED STATES

## Abstract

High tibial osteotomy (HTO) has been widely used for clinical treatment of osteoarthritis of the medial compartment of the knee, and both opening-wedge and closing-wedge HTO are the most commonly used methods. However, it remains unclear which technique has better clinical and radiological outcomes in practice. To systematically evaluate this issue, we conducted a comprehensive meta-analysis by pooling all available data for the opening-wedge HTO and closing-wedge HTO techniques from the electronic databases including PubMed, Embase, Wed of Science and Cochrane Library. A total of 22 studies encompassing 2582 cases were finally enrolled in the meta-analysis. There was no significant difference regarding surgery time, duration of hospitalization, knee pain VAS, Lysholm score and HSS knee score (clinical outcomes) between the opening-wedge and closing-wedge HTO groups (*P* > 0.05). However, the opening-wedge HTO group showed wider range of motion than the closing-wedge HTO group (*P* = 0.003). Moreover, as for Hip-Knee-Ankle angle and mean angle of correction, no significant difference was observed between the opening-wedge and closing-wedge HTO groups (*P* > 0.05), while the opening-wedge HTO group showed greater posterior tibial slope angle (*P* < 0.001) and lesser patellar height than the closing-wedge HTO group (*P* < 0.001). On light of the above analysis, we believe that individualized surgical approach should be introduced based on the clinical characteristics of each patient.

## Introduction

Knee osteoarthritis (OA) is a common and multi-factorial arthritic disorder, which may lead to joint dysfunction, *i*.*e*. reduction of joint motion and physical disability, as a result of degeneration, destruction, and loss of articular cartilage of knee joint [1]. Most patients with knee OA would develop varus deformity, and varus malalignment further overloads the medial tibiofemoral compartment, causing degenerative changes in the articular cartilage at last. Both conservative methods and surgical treatment were used for treatment of pain and dysfunction in knee OA. Surgical treatment included various techniques, *i*.*e*. high tibial osteotomy (HTO), arthroscopic surgery and total knee arthroplasty. Among them, HTO is the most frequently used method in young and more active patients [2].

In previous studies, various techniques of HTO were documented and evaluated. Among them, closing-wedge HTO and opening-wedge HTO, which were stabilized by a locking plate [3, 4], were two of the most frequently used techniques. For the closing-wedge HTO, a wedge-shaped cut is made in the lateral; while for the opening-wedge HTO, the medial tibia is cut. Many previous studies have compared the advantages and disadvantages between the closing-wedge and the opening-wedge HTO [5–7]. For example, the closing-wedge HTO was more advantageous in patients with a medial compartment OA with varus deformity for decrease of the posterior tibial slope [8]. However, several disadvantages have been also reported for the closing-wedge HTO, such as the need for fibular osteotomy, bone stock loss and nervous system complications [4, 5]. Different from the closing-wedge HTO, the opening-wedge HTO which has been performed more recently and doesn’t need a fibula osteotomy, caused fewer co-morbidities related to the fibular ostetomy [4]. Compared to the closing-wedge HTO, the opening-wedge HTO has several advantages, *i*.*e*. easier adjustment of alignment correction, more rapid rehabilitation, as reported previously [9, 10].

In detail, the advantages, disadvantages and sometimes complications were compared between the opening-wedge and closing-wedge HTO [7, 8, 11]; however, the results remained inconsistent. In the present study, we performed a comprehensive meta-analysis of the clinical and radiological outcomes between the opening-wedge and closing-wedge HTO, to explore whether the opening-wedge HTO was superior to the closing-wedge HTO. Our study would provide reasons for clinical guidance of surgical choice.

## Materials and methods

### Eligibility criteria

Eligible studies must meet the following criteria: 1) articles published in English and peer-reviewed journals; 2) randomized controlled trials (RCTs) and non-RCTs design comparing clinical and/or radiographic outcomes between closing-wedge HTO and opening-wedge HTO; 3) patient samples were independent from each other (if samples from different studies overlapped, only the studies with the largest sample size were included).

Animal study, review, commentary and meeting abstracts without detailed and novel data were excluded from the meta-analysis.

### Search strategy

We searched eligible studies for this meta-analysis from PubMed (http://www.ncbi.nlm.nih.gov), SCOPUS (http://www.scopus.com), EMBASE (http://www.elsevier.com/online-tools/embase), ISIWeb of Knowledge (http://apps.webofknowledge.com/) and Cochrane Library (http://www.cochranelibrary.com/). Search terms used in the title, abstract and MeSH term included (“Osteotomy” OR ‘‘Tibial”) and (“Closed” OR “Closing”) and (“Open”). After the initial electronic search, eligible studies were screen carefully for other eligible studies. Studies published in English before the 1st of April 2016 were considered.

### Data extraction

For each eligible study, two independent investigators extracted the following data: 1) first author and publication year; 2) type of study design; 3) sample size, age and gender distribution; 4) follow-up periods; 5) clinical and radiographic outcomes; 6) other data if essential. Any disagreement of data was subject to the third author. Moreover, if essential data was not available directly from the manuscript, the corresponding author was contacted, or we calculated them, i.e. standard deviations and confidence intervals, from already provided data.

In detail, clinical outcomes included, but not limited to, time course of surgical operation, duration of hospital stays, various sorts of knee scoring (Hospital for Special Surgery (HSS) knee score, knee pain visual analog score, Lysholm knee score and Wallgren-Tegner score), knee range of motion (ROM), as well as patient satisfaction (score). Radiographic outcomes included, but not limited to, mean angle of correction, hip–knee–ankle (HTA) angle, posterior tibial slope angle, and patellar height (PH).

### Assessment of methodological quality

The revised Jadad scale by the Cochrane Non-randomized Studies Methods Working Group were used to assess methodological quality [12]. A total of 7 points were assigned to this scoring method, including the randomization process (2 points), allocation concealment (2 points), appropriateness of blinding (2 points), and a description of dropouts and withdrawals (1 point). A score of 4–7 points indicated high quality, and 0–3 indicating poor quality. Two investigators independently extracted the data using a standardized form. The final score was based on the consensus assessment from two investigators. If disagreements persisted, a third author was consulted.

### Statistical analysis

The Stata 12.0 statistical software package (http://www.stata.com/) and the Review Manager 5.3 software (The Cochrane Collaboration) were applied to conduct publication bias analysis, meta-analysis and sensitivity analysis. Potential publication bias was determined by both the Egger regression test for a funnel plot [13] and the Begg–Mazumdar test, which is based on Kendall’s-τ [14]. For dichotomous variables, the odds ratio (OR) with 95% confidence interval (95% CI) was derived for each outcome; for continuous variables, we calculated the standardized mean difference and 95% CI. We performed the meta-analysis using a fixed-effect model if no significant heterogeneity was present; otherwise, a random-effect model was applied. To assess heterogeneity between studies, we performed a chi-square test and estimated the I^2^ statistic. For all analyses, *P* < 0.05 was considered statistically significant. The study was approved by the ethics committee of Lishui People’s Hospital of Wenzhou Medical University. All the protocols and experimental procedures were in accordance with the Declaration of Helsinki and other international rules **(S1 Table)**.

## Results

### Literature search and eligible studies

**Fig 1:**
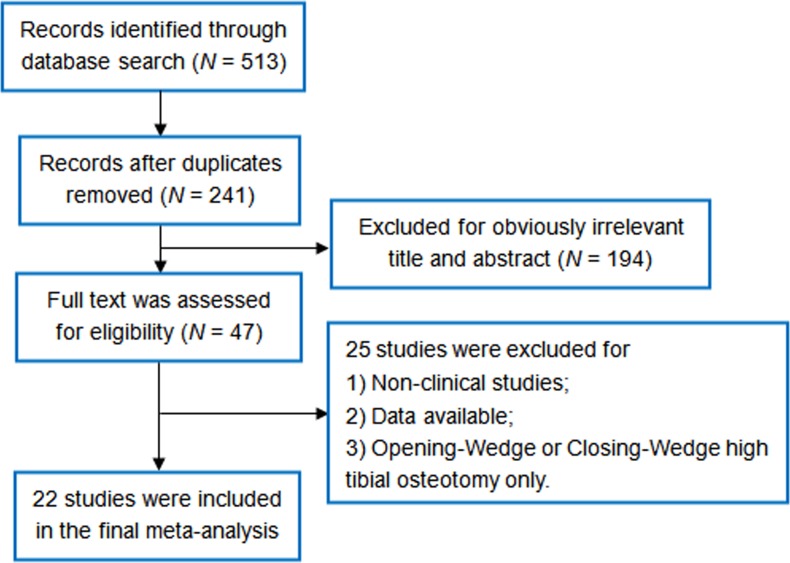
Flow diagram of the identification and selection of the studies included in this meta-analysis.

According to our literature search approaches, a total of 513 references were indexed. After excluding 487 studies for reasons specified in **Fig 1 and S1 Fig**, a total of 22 studies (7 RCTs and 15 non-RCTs) encompassing 2582 cases, including 1274 knees which were underwent opening-wedge HTO and 1308 ones with closing-wedge HTO operation, were finally enrolled in the final meta-analysis [5–8, 10, 15–31]. **Table 1**listed the detailed information of recruited studies for this meta-analysis, i.e. sample size, age and gender proportion, fixation approaches, etc. It is worth note that four studies [29, 32–34] were removed from the current meta-analysis due to sample duplication within other studies [17, 28, 31].

**Table 1:** Characteristics of the included studies.

Author	Year	Study design	Opening-wedge HTO				Closing-wedge HTO				Quality score
			N	Age	Gender (M/F)	Fixation method	N	Age	Gender (M/F)	Fixation method	
van Egmond et al.	2016	RCT	25	47.1 (8.5)	15/10	Locked plate	25	50.3 (7.4)	16/9	Locked plate	4
Duivenvoorden et al.	2015	RCS	112	48.7 (10.1)	73/39	Puddu and Tomofix plate	354	49.4 (9.0)	203/151	Three different staples	6
Nerhus et al.	2015	RCT	35	NA	NA	Staples	35	NA	NA	Puddu titanium plate	4
Deie et al.	2014	RCS	9	57.5 (6.0)	3/6	Plate and screws	12	57.8 (6.0)	3/9	Plate and screws	6
Portner et al.	2014	RCS	26	43.9 (8.48)	20/6	Plate and screws	18	46.5 (5.17)	15/3	Staples	6
Duivenvoorden et al.	2014	RCT	36	49.9 (7.9)	24/12	Puddu plate	45	49.5 (9.2)	27/18	Staples	6
Amzallag et al.	2013	PCS	224	53.6 (8.6)	NA	NA	97	49.7 (10.3)	NA	NA	8
Tabrizi et al.	2013	PCS	21	36.5 (8.1)	13/3	Plates	21	35.1 (9.7)	12/4	L or T plates	7
Bae et al.	2013	RCS	30	56.3 (7.5)	2/25	Puddu plate	78	58.8 (7.5)	4/70	Miniplate staple	6
Song et al.	2012	RCS	50	57.9	10/40	Wedge plates	50	60.1	12/38	Stepped staples	7
Ducat et al.	2012	PCS	210	52 (9)	NA	NA	92	49.7 (10.3)	NA	NA	6
Magnussen et al.	2011	RCS	32	54	22/10	Tomofix plate	32	59	21/9	Blade and screws	8
Song et al.	2010	RCS	90	51	21/69	Aescula plates	104	57	16/88	Staples	6
Gaasbeek et al.	2010	RCT	25	47.0 (8.5)	14/11	Locked plate	25	49.8 (8.4)	16/9	Locked plate	4
Hankemeier et al.	2010	RCS	35	44	NA	Fixed-angle plates	26	53	NA	Screw-plate	5
Luites et al.	2009	RCT	23	53	NA	TF plates and screws	19	53	NA	TF plates and screws	5
van den Bekerom et al.	2008	PCS	20	52	10/10	Puddu Plate	20	52	9/11	AO/ASIF L-plate	7
Schaefer et al.	2008	RCS	90	46	NA	T-Clamp	66	47	NA	Wedge Blount’s staples	7
El-Azab et al.	2008	RCS	60	NA	NA	NA	60	NA	NA	NA	6
Brouwer et al.	2005	RCT	45	49.6	32/13	Plates	47	50.8	27/20	L-plates	4
Hoell et al.	2005	RCS	51	52.1 (8.4)	32/19	Puddu plates	57	46.4 (8)	40/17	Coventry	
Magyar et al.	1999	RCT	25	55	NA	External fixation	25	55	NA	Staples	5

HTO, high tibial osteotomy; RCT, ndomized controled trial; RCS, retrospective cohort study; PCS, prospective cohort study; NA, not available

### Clinical outcomes

As for clinical outcomes, the following several aspects were compared between opening-wedge and closing-wedge HTO, *i*.*e*. surgery time, duration of hospital stays, HSS knee score (> 1 year follow-up), knee pain VAS, range of motion (flexion angle) and Lysholm knee score. Other parameters, including patient satisfaction (score), Wallgren-Tegner score, Insall-Salvati index and complete weight bearing (day), were not pooled in our meta-analyses due to limited studies (*N* < 2) or sample size. Notably, since no significant difference was observed for pre-operative characteristics between the opening-wedge and closing-wedge HTO groups, only the post-operative values of clinical outcomes were compared.

As shown in **Table 2**, there was no significant difference for surgery time (SMD = -0.18, 95% CI = -1.24–0.88, *P* = 0.741) [5, 19, 24, 30] and duration of hospitalization (SMD = -0.87, 95% CI = -2.09–0.36, *P* = 0.166) [5, 24, 31] as indicated by the random-effect model.

**Table 2:** Overview of meta-analysis results.

Index	Studies	Sample size	Heterogeneity (I^2^/*P*-value)	Effect			Model
				Z-score	P-value	OR or SMD (95% CI)	
**Clinical outcomes**							
Surgical time	4	209/457	95.9% (<0.001)	0.33	0.741	-0.18 (-1.24, 0.88)	Random
Duration of hospitalization	3	162/404	94.8% (<0.001)	1.38	0.166	-0.87 (-2.09, 0.36)	Random
HSS knee score	4	201/224	0.0% (0.607)	1.81	0.07	0.18 (-0.01, 0.37)	Fixed
Knee pain VAS	5	154/161	0.0% (0.436)	0.00	0.999	0.00 (-0.22, 0.22)	Fixed
Lysholm score	4	120/122	0.0% (0.773)	1.43	0.152	0.19 (-0.07, 0.44)	Fixed
Range of motion (flexion angle)	3	152/164	0.0% (0.777)	**2.94**	**0.003**	0.33 (0.11, 0.56)	Fixed
**Radiological outcomes**							
Hip-Knee-Ankle angle	7	459/397	82.8% (<0.001)	0.81	0.415	-0.15 (-0.52, 0.22)	Random
Mean angle of correction	8	310/327	79.1% (<0.001)	0.00	0.998	0.00 (-0.36, 0.36)	Random
Posterior tibial slope angle	11	663/581	95.0% (<0.001)	**4.27**	**<0.001**	1.31 (0.71, 1.91)	Random
PH: Caton index	2	249/122	0.0% (0.408)	**7.88**	**<0.001**	-0.92 (-1.15, -0.56)	Fixed
PH: Insall-Salvati index	3	82/64	17.9% (0.296)	**2.18**	**0.029**	-0.36 (-0.67, -0.04)	Fixed
PH: Blackburne-Peel ratio	2	95/97	35.0% (0.215)	0.88	0.377	-0.13 (-0.41, 0.16)	Fixed

VAS, Visual Analogue Scale; PH, patellar height; OR, odds ratios; SMD, standardized mean differences

As the main characteristics of clinical outcome, HSS knee score [6, 17, 21, 31], knee pain VAS [7, 17, 24, 25, 29] and Lysholm score [19, 25, 30, 31] were assessed in four, five and four studies, respectively. Our meta-analysis indicated that there was no significant difference regarding knee pain VAS (SMD = 0.00, 95% CI = -0.22–0.22, *P* = 0.999) and Lysholm score (SMD = 0.19, 95% CI = -0.07–0.44, *P* = 0.152), between the opening-wedge and closing-wedge HTO groups using the fixed-effect model (*P* > 0.05); while slightly but not significantly higher HSS knee score was observed in the opening-wedge HTO group, when compared with the closing-wedge HTO group (SMD = 0.18, 95% CI = -0.01–0.37, *P* = 0.07) using the fixed-effect model (I^2^ = 0.7%, *P* = 0.388) **(Table 2)**.

**Fig 2:**
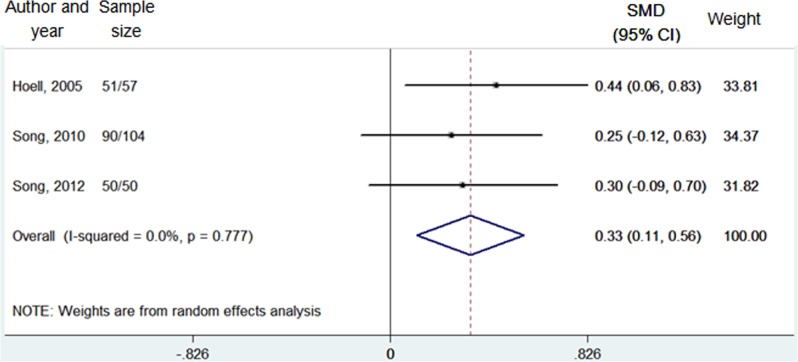
Forest plot of range of motion (flexion angle) between the opening-wedge and closing-wedge HTO group. Note: sample size represented N opening-wedge HTO/N closing-wedge HTO; SMD, standardized mean difference.

Interestingly, significant difference was observed regarding the range of motion (flexion angle) (*P* = 0.003) between the opening-wedge and closing-wedge HTO groups by the fixed-effect model (I^2^ = 0.0%, *P* = 777) **(Table 2, Fig 2)**, and the opening-wedge HTO group showed wider range of motion than the closing-wedge HTO group (SMD = 0.33, 95% CI = 0.11–0.56) [6, 21, 30].

### Radiological outcomes

#### Hip-Knee-Ankle angle

A total of 7 studies encompassing 459 cases with opening-wedge HTO and 397 knees with closing -wedge HTO were included for HKA angle analysis [6, 8, 10, 15, 17, 18, 20]. As shown in **Table 2**, there was no significant difference regarding the HKA angle after surgical operation between the opening- and closing-wedge HTO groups (SMD = -0.15, 95% CI = -0.52–0.22, *P* = 0.415) using the random-effect model (I^2^ = 82.8%, *P* < 0.001) **(S2 Fig)**.

#### Mean angle of correction

There were 8 studies including 637 knees with analysis of mean HKA angle of correction [7, 8, 10, 20, 24, 27, 29, 31]. After pooling all the data, we observed no significant difference between knees undergoing opening-wedge HTO and those undergoing opening-wedge HTO (SMD = 0.00, 95% CI = -0.36–0.36, *P* = 0.998) by the random-effect model (I^2^ = 79.1%, *P* < 0.001) **(Table 2, S3 Fig)**.

#### Posterior tibial slope angle

**Fig 3:**
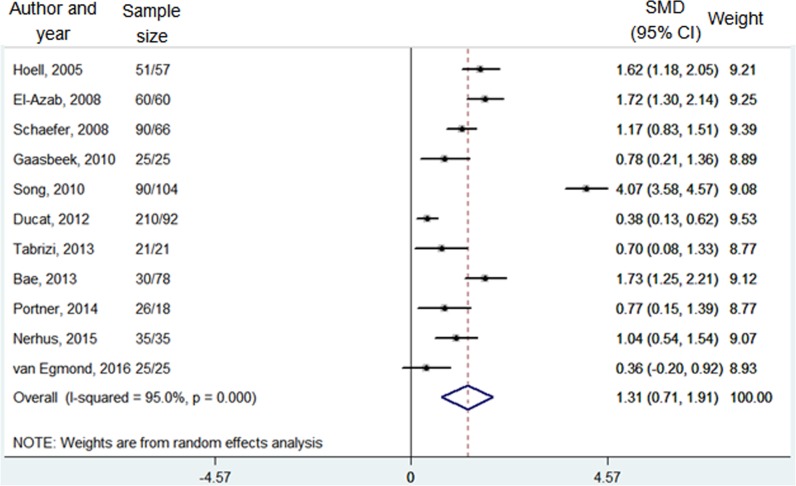
Forest plot of posterior tibial slope angle between the opening-wedge and closing-wedge HTO grou. Note: sample size represented N opening-wedge HTO/N closing-wedge HTO; SMD, standardized mean difference.

Data on 663 knees with opening-wedge HTO and 581 knees with closing-wedge HTO from a total of 11 studies were pooled together regarding the posterior tibial slope angle [6–8, 16, 19, 20, 22, 24, 27, 28, 30]. Meta-analysis with random-effect model (I^2^ = 95.0%, *P* < 0.001) showed that there was a significantly greater posterior tibial slope angle in the opening-wedge HTO cases compared to the closing-wedge HTO ones (SMD = 1.31, 95% CI = 0.71–1.91, *P* < 0.001) **(Table 2 and Fig 3)**.

#### Patellar height

**Fig 4:**
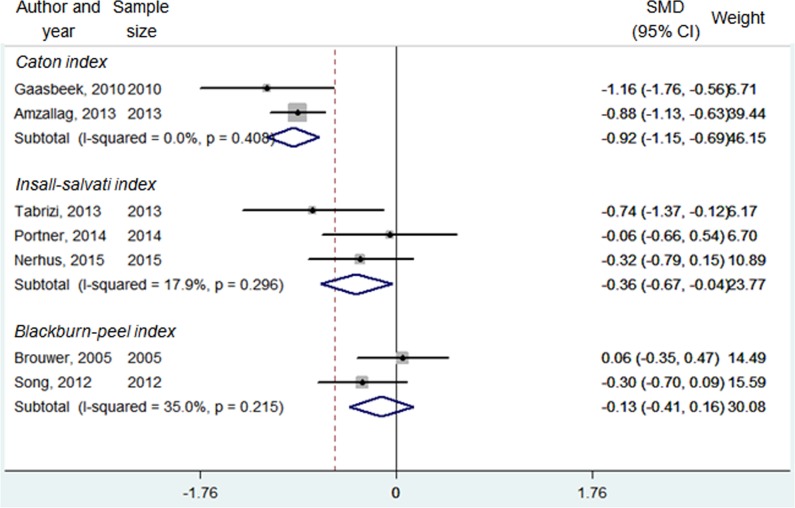
Forest plot of patellar height (Caton index, Insall-Salvati index and Blackburne-Peel index) between the opening-wedge and closing-wedge HTO group. Note: sample size represented N opening-wedge HTO/N closing-wedge HTO; SMD, standardized mean difference.

Besides, three indicators of patellar height were analyzed, including Caton index [18, 24], Insall-Salvati index [8, 16, 19] and Blackburne-Peel ratio [21, 29]. As shown in **Table 2 and Fig 4**, there was a significantly greater patellar height measured by the Caton index (SMD = -0.92, 95% CI = -1.15 –(-0.56), *P* < 0.001) and Insall-Salvati index (SMD = -0.36, 95% CI = -0.67 –(-0.04), *P* < 0.001) in the closing-wedge HTO group when compared with the opening-wedge HTO group. However, when measured by Blackburne-Peel ratio, no significant difference was observed between these two groups.

## Discussion

In the present study, we performed a comprehensive meta-analysis of clinical and radiographic outcomes between the opening-wedge and closing-wedge HTO groups. For most of the included indexes, no significant difference was observed between these two groups. However, for some outcomes, *i*.*e*., postoperative range of motion (flexion angle), posterior tibial slope angle and patellar height, there was indeed significant difference between the opening-wedge and closing-wedge HTO groups.

As for clinical outcomes, only the range of motion (flexion angle) showed significant difference between the opening-wedge HTO and closing-wedge HTO groups (*P* = 0.003), and the former exhibited wider range of motion than the latter (SMD = 0.33, 95% CI = 0.11–0.56). As we know, a portion of patients undergoing the opening-wedge HTO were required to receive range-of-motion exercise during the early postoperative period, and in Song *et al*. study [6, 21], all the opening-wedge HTO patients received the range-of-motion exercise, which was not the case for the closing-wedge group. In our meta-analysis, two studies from Song *et al*. constitute the largest portion of the overall sample size, which might lead to biased result regarding the range of motion (flexion angle).

As for the patellar height, we pooled data from three distinct measuring methods, including Caton index, Insall-Salvati index and Blackburne-Peel index. Our analyses showed that the patellar height, as measured by Caton index and Insall-Salvati index, was significantly decreased after the opening-wedge HTO than that after the closing-wedge HTO. Careful examination of the raw data from previous studies, we observed that the patellar height was decreased after the opening-wedge HTO, while increased after the closing-wedge HTO. The possible explanation of this controversial result was that the opening-wedge HTO may lower the tibial tuberosity leading to decreased patella height, while the closing-wedge HTO could elevate the tibial tuberosity as a result of a proximalisation of the proximal tibia [35]. Different from our meta-analysis, Takeuchi *et al*. found that patellar height was maintained after opening-wedge HTO and speculated that this result was due to range-of-motion exercises during the early postoperative period [36]. However, in Song *et al*. study, in which range-of-motion exercise was recommended after opening-wedge HTO, most patients showed a significant decrease in patellar height after surgery as well, suggesting that range-of-motion exercise was not the main cause of decrease in patellar height [21]. Intriguingly, slight but not significant reduction of patella height as measured by Blackburne-Peel index was observed in the opening-wedge HTO group when compared with that of the closing-wedge group (*P* = 0.377). This inconsistency may be resulted from the limited sample size, which could cause publication bias and insufficient statistical power.

Moreover, the result of the meta-analysis showed that posterior tibial slope angle after opening-wedge HTO was significantly increased than that after closing-wedge HTO (SMD = 1.31, 95% CI = 0.71–1.91). Similar with the patellar height, the posterior slope was increased following the opening-wedge HTO, while decreased after the closing-wedge HTO. It is one frequently reported problem associated with the opening-wedge HTO that posterior tibial slope might be increased. Several theories have been put forward to explain the etiology of changes of posterior tibial slope following HTO. The most widely accepted theory is that the unique anatomic geometry of the proximal tibia is responsible for the change of posterior tibial slope angle following HTO [37]. This geometric shape requires a smaller opening gap at the anterior part of the osteotomy site than the posteromedial part, as the former is closer to the hinge point of osteotomy if the posterior tibial slope angle remains unchanged [38].

Several limitations should be presented to interpret the present results. Firstly, different surgical approaches were included and mixed in the current meta-analysis, which may confound the meta-analysis result. Secondly, though most of the included studies were of high-quality, a small portion of the included studies were of low-quality as indicated by the revised Jaded scale (**Table 1)**. Insufficient method description, low reliability of radiographic results, and study design in these studies might lead to between-group heterogeneity. Thirdly, although all the eligible studies were included in this meta-analysis, the sample size was relatively small, which may induce type-II statistic error. Updated meta-analysis of this issue is essential for a more solid conclusion in future.

## Conclusions

Except for the range of motion (flexion angle), posterior tibial slope angle and patellar height, both the opening-wedge and closing-wedge HTO had satisfactory and comparable clinical and radiographic outcomes. On light of the above analysis, we believe that individualized surgical approach should be introduced based on the clinical characteristics of each patient.

## Supporting information

S1 Table PRISMA 2009 checklist. (DOC)

S1 Fig PRISMA 2009 flow diagram. (DOC)

S2 Fig Forest plot of HKA angle between the opening-wedge and closing-wedge HTO groups. Note: sample size represented N opening-wedge HTO/N closing-wedge HTO; SMD, standardized mean difference.
(DOC)

S3 Fig Forest plot of mean angle of correction between the opening-wedge and closing-wedge HTO groups. Note: sample size represented N opening-wedge HTO/N closing-wedge HTO; SMD, standardized mean difference.
(DOC)
