# Supplementary material for: Comparison of clinical and radiological outcomes between opening-wedge and closing-wedge high tibial osteotomy: A comprehensive meta-analysis
Source: PLoS One. 2017 Feb 9;12(2):e0171700. doi: 10.1371/journal.pone.0171700 (PMC5300239; doi:10.1371/journal.pone.0171700)
Supplement: S1 Fig — (DOC) [file pone.0171700.s002.doc]

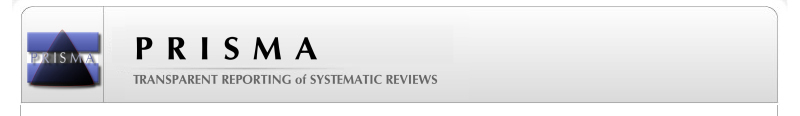
**S1 Fig PRISMA 2009 Flow Diagram**

**Screening**

**Included**

**Eligibility**

**Identification**

Records identified through database searching
(n = 513 )

Additional records identified through other sources
(n = 0 )

Records after duplicates removed
(n = 241 )

Records screened
(n = 241 )

Records excluded
(n = 194 )

Full-text articles assessed for eligibility
(n = 47 )

Full-text articles excluded, with reasons
(n = 25 )

Studies included in qualitative synthesis
(n = 22 )

Studies included in quantitative synthesis (meta-analysis)
(n = 22 )
