# Supplementary material for: Comparison of clinical and radiological outcomes between opening-wedge and closing-wedge high tibial osteotomy: A comprehensive meta-analysis
Source: PLoS One. 2017 Feb 9;12(2):e0171700. doi: 10.1371/journal.pone.0171700 (PMC5300239; doi:10.1371/journal.pone.0171700)
Supplement: S2 Fig — Note: sample size represented N opening-wedge HTO/N closing-wedge HTO; SMD, standardized mean difference. (DOC) [file pone.0171700.s003.doc]

**S2 Fig Forest plot of HKA angle between the opening-wedge and closing-wedge HTO groups**


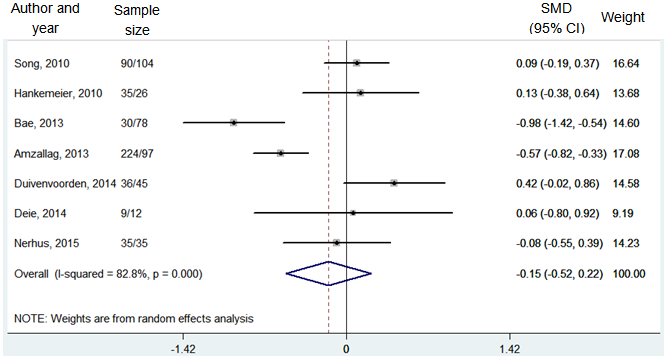


Note: sample size represented N opening-wedge HTO/N closing-wedge HTO; SMD, standardized mean difference.
